# Experimental Dissection of the Lytic Replication Cycles of Herpes Simplex Viruses *in vitro*

**DOI:** 10.3389/fmicb.2018.02406

**Published:** 2018-10-11

**Authors:** Francisco J. Ibáñez, Mónica A. Farías, Maria P. Gonzalez-Troncoso, Nicolás Corrales, Luisa F. Duarte, Angello Retamal-Díaz, Pablo A. González

**Affiliations:** Millennium Institute on Immunology and Immunotherapy, Departamento de Genética Molecular y Microbiología, Facultad de Ciencias Biológicas, Pontificia Universidad Católica de Chile, Santiago, Chile

**Keywords:** life cycle herpes simplex viruses, replication cycle herpes simplex virus, herpes simplex virus infection steps, antivirals, acyclovir, antiviral drugs

## Abstract

Herpes simplex viruses type 1 and type 2 (HSV-1 and HSV-2) produce lifelong infections and are highly prevalent in the human population. Both viruses elicit numerous clinical manifestations and produce mild-to-severe diseases that affect the skin, eyes, and brain, among others. Despite the existence of numerous antivirals against HSV, such as acyclovir and acyclovir-related analogs, virus variants that are resistant to these compounds can be isolated from immunosuppressed individuals. For such isolates, second-line drugs can be used, yet they frequently produce adverse side effects. Furthermore, topical antivirals for treating cutaneous HSV infections usually display poor to moderate efficacy. Hence, better or novel anti-HSV antivirals are needed and details on their mechanisms of action would be insightful for improving their efficacy and identifying specific molecular targets. Here, we review and dissect the lytic replication cycles of herpes simplex viruses, discussing key steps involved in cell infection and the processes that yield new virions. Additionally, we review and discuss rapid, easy-to-perform and simple experimental approaches for studying key steps involved in HSV replication to facilitate the identification of the mechanisms of action of anti-HSV compounds.

## Introduction

Herpes simplex viruses type 1 (HSV-1) and type 2 (HSV-2) are two *Alphaherpesvirinae* viruses that are highly prevalent in the human population and are known to produce numerous clinical manifestations after the infection of different tissues within the host. While the world prevalence for HSV-1 nears 67%, estimates for HSV-2 fluctuate between 11 and 20% (http://www.who.int) (Looker et al., 2015). Infections with HSVs mainly occur after these viruses have gained contact with the mucosae or micro-lesions in skin epithelia; dissemination in turn ensues from oral and genital secretions (Kaufman et al., 2005). Similar to other herpesviruses, HSV infections are lifelong and generally asymptomatic, yet the viruses can be shed from infected individuals independent of the occurrence of clinical manifestations (Wald et al., 2000). Additionally, HSVs can infect neuronal prolongations enervating peripheral tissues and establish latency in these cells, namely in the trigeminal ganglia and dorsal root ganglia of the sacral area from where they can sporadically reactivate (Gillgrass et al., 2005; Margolis et al., 2007; Huang et al., 2011).

Despite numerous efforts invested in creating prophylactic formulations against HSV-1 and HSV-2, at present there are no vaccines against these viruses. An important effort consisting on a subunit protein-based formulation with the viral glycoprotein D as the main viral antigen combined with adjuvants, was reported to yield disappointing results after a phase 3 clinical trial (Kwant and Rosenthal, 2004; Belshe et al., 2012). Because of the lack of a vaccine against HSVs, antivirals are frequently used as a resource to treat the clinical manifestations that these viruses produce. While acyclovir and acyclovir-derived nucleoside analogs can prevent severe HSV infections, their absorption by the organism is somewhat limited and when applied in the form of topical creams for treating skin infections they usually show poor efficacy (Spruance et al., 1990). Additionally, the effectiveness of acyclovir and other commonly used anti-HSV antivirals is sometimes compromised by the occurrence of drug-resistant variants, which mostly arise in immunocompromised individuals; these antiviral-resistant isolates will require second-line drugs for their treatment, yet these compounds often produce significant adverse effects (Ziyaeyan et al., 2007; Suazo et al., 2015b). Therefore, antivirals that can effectively block the replication cycle of HSVs with few-to-none side effects are needed. Furthermore, understanding the mechanisms of action of such anti-HSV drugs could help design better antiviral compounds and potentially contribute at identifying additional drugs against HSVs and other herpesviruses. Our present knowledge on the molecular processes associated to the replication cycles of HSVs and their capacity to overcome cellular antiviral mechanisms provides excellent opportunities for identifying the mechanisms of action of antiviral compounds against these viruses (Suazo et al., 2015a).

Here, we review and discuss key steps involved in the lytic replication cycles of HSVs *in vitro*, namely processes related to virus binding to the cell surface, capsid entry into the cytoplasm, capsid migration within the cytoplasm together with viral genome delivery into the nucleus, viral gene expression, genome replication, virion assembly, and virus egress. Along the revision of these processes, we discuss simple and rapid experimental approaches that can be performed for assessing each of these steps in cells such as fibroblasts and epithelial cells. We also highlight anti-HSV compounds with known mechanisms of action and discuss experimental assays that can be used to narrow down the specific steps at which antivirals interfere with the lytic replication cycle of HSVs. Once the viral replication step that is blocked by the antiviral is recognized, more precise experimental approaches can be performed to examine the exact molecular factors involved.

## Antivirals against HSVs

One of the first antiherpetic nucleoside analogs reported to be effective in the clinic is 5-iodo-2′-deoxyuridine (IDU) (Prusoff, 1959; Kaufman, 1962; Kaufman et al., 1962), which was then followed by vidarabine, the earliest drug to be used for systemic treatment of HSV infection (O'Day et al., 1976). At the end of the 70's, acyclovir an acyclic nucleoside analog which targets the herpesvirus DNA polymerase was introduced and nearly 50 years after it is still in use to treat HSV infections, mainly because of its safety record and acceptable efficacy (Elion et al., 1977). However, due to its low intestinal absorption variations of acyclovir were later developed (penciclovir, valacyclovir, and famciclovir), which simplify the treatment by reducing the frequency with which the antivirals needs to be taken during the day (Poole and James, 2018). Although all of these drugs significantly impact HSV replication in the host, they do not completely block virus shedding or clinical manifestations (Wald et al., 2006). Furthermore, for some clinical presentations such *herpes labialis* topical acyclovir only reduces in 1–2 days the length of HSV skin lesions, which can extend up to 10–14 days in primary infections and 7–10 days during recurrences (Moomaw et al., 2003; Arduino and Porter, 2008). Additionally, HSV isolates that are resistant to these drugs can be isolated from immunosuppressed individuals infected with these viruses, in which mutations are usually concentrated in the DNA polymerase (*U*_*L*_*30*) and thymidine kinase (*U*_*L*_*23*) genes (Sauerbrei et al., 2011) (reviewed in Suazo et al., 2015b). Second-line drugs, such as foscarnet which is a pyrophosphate analog and cidofovir, which is an acyclic phosphonate nucleotide analog also target the viral polymerase, yet at different sites than acyclovir and its analogs, and are approved for the treatment of acyclovir-resistant HSV infections in immunocompromised patients (De Clercq, 2013). Although effective against acyclovir-resistant isolates, these two drugs need to be administered intravenously and require medical supervision because of possible nephrotoxicity and kidney damage. Hence, novel drugs against herpes simplex viruses are welcome and needed. At present, numerous drugs, which are diverse in kind, such as synthetic drugs, recycled FDA-approved drugs and natural extracts display antiviral activity against HSV, with some of them in pre-clinical stages and others in advanced clinical trials (Hassan et al., 2015; Chaudhuri et al., 2018; Poole and James, 2018). Importantly, many of these drugs target viral components and processes that are different from the viral polymerase, which provides opportunities for combined drug therapies, such as those used against HIV (Andrei et al., 2011). As an example, several new antivirals target the viral DNA helicase/primase complex, such as BAY 57-1293 (Kleymann et al., 2002), ASP2151 (Katsumata et al., 2013), and AIC316 (Vere Hodge and Field, 2013), among others. Other antivirals target either other viral determinants or modulate host factors. For instance, some anti-HSV compounds modulate host cyclin-dependent kinases (CDKs) (Schang et al., 2002), the phosphorylation of AKT (Jaishankar et al., 2018), or the proteasome and NF-κB activation (La Frazia et al., 2006). How antivirals against HSV impact the replication of these viruses is discussed below together with a review of the key steps involved in the life cycle of these viruses. Importantly, determining the mechanism of action of antivirals is important for identifying other potential drugs with similar mode of action and to foresee potential toxic effects over healthy cells. Besides, knowing the mechanism of action of a drug is nowadays almost a prerequisite for clinical development.

Although the current review focuses on the lytic replication cycles of HSVs and antivirals that block their related processes, it is important to note that novel therapeutic strategies are also being developed to attack these viruses in the latent phases. Noteworthy, CRISPR/Cas9 has been harnessed to target several HSV genes both, required for latent and lytic infection and has been shown to almost completely suppress viral infection *in vitro*. Although the strategy in this study was ineffective in targeting quiescent HSV-1, it did abrogate replication of reactivated HSV-1 (van Diemen et al., 2016). On the other hand, targeted endonucleases encoded within adeno-associated virus (AAV) aimed at essential HSV genes was shown to induce mutations in latent HSV genomes *in vivo* in a model of latent HSV infection (Aubert et al., 2016). The use of CRISPR/Cas in targeting herpesviruses is reviewed in two recent articles (van Diemen and Lebbink, 2017; Chen et al., 2018).

**Figure 1:**
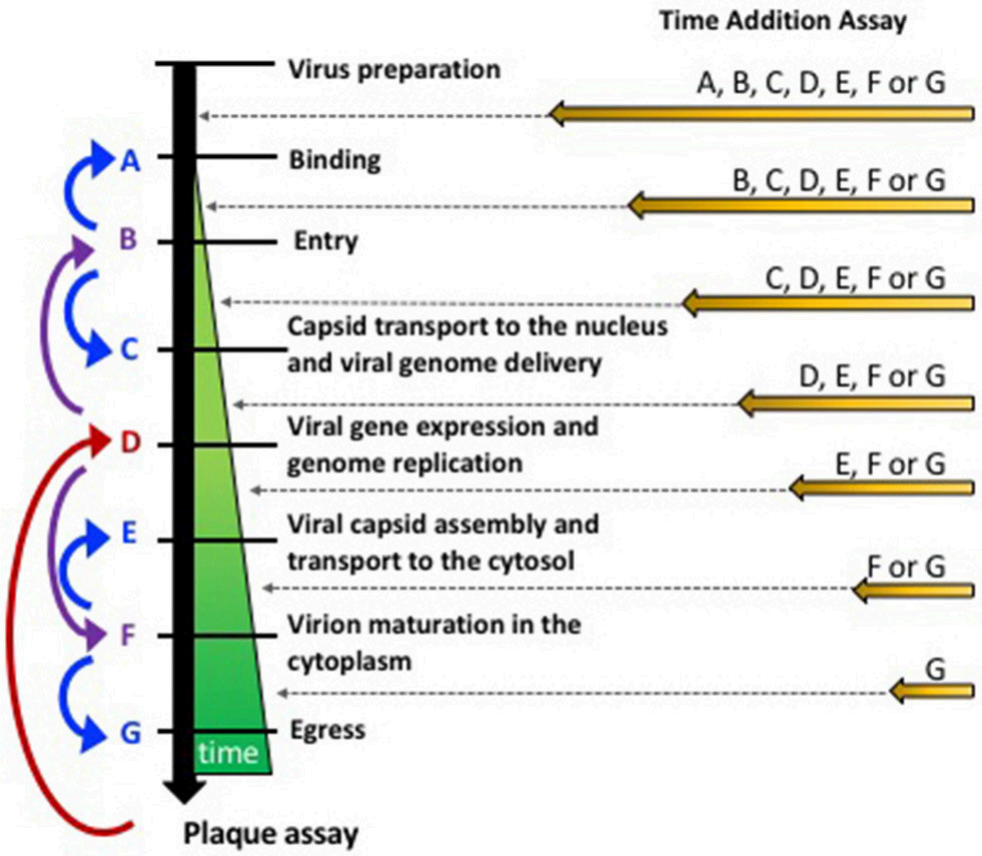
Approaches for assessing the mode of action of anti-HSV compounds. **(Left)** Strategy to sequentially assess different steps of the replication cycle of HSVs starting with the tittering of PFUs at the end of the life cycle of these viruses in susceptible cells. Once an antiviral effect has been evidenced by tittering PFU in the supernatants, viral gene expression or genome replication can be performed (red arrow) and establish if the mode of action of the drug is upstream or downstream of this process (purple arrow). Successive experiments seek to narrow down the step involved by assessing additional middle step between adjacent processes (blue arrows). **(Right)** Time-of-Drug Addition approach, which consists on assessing how long after infection can the addition of the antiviral be postponed to obtain an antiviral effect (reduction in PFUs).

A common approach for identifying the mechanism of action of antiviral drugs that hamper virus replication is performing “Time-of-Drug Addition” assays *in vitro*, which can help narrow down the mode of action of such compounds in just few days (Figure 1) (Daelemans et al., 2011). Overall, these assays allow determining how long after infection can the antiviral be added to the culture before losing its antiviral effect. A practical and easy readout for such assays is PFU yield, assessed by titering the supernatants of the treated cells. Importantly, these assays must be performed in the time frame of one virus cycle and tight timing schedules need to be implemented for consistency and narrowing down the particular virus replication step involved. Because the replication cycle of a virus may slightly vary depending on the cell type, generating empiric data on the timing of each step may be needed, which can be performed using inhibitors with known mechanisms of action (Daelemans et al., 2011).

Alternatively, the mechanism of action of an antiviral can be determined by dissecting the virus replication cycle into key steps, which can be sequentially assessed (Figure 1). In such scenario, the antiviral activity of a drug should be first determined by assessing the output of infectious viral particles. If an antiviral effect is observed, an intermediate step in the replication cycle of the virus can be evaluated, such as viral gene expression or genome replication (Figure 1, step D). From there on, experimental assays that further evaluate intermediate steps in the virus replication cycle will ultimately lead to the identification of a particular step that is hampered by the antiviral drug. A specific step in the replication cycle can then be further dissected to finally identify the molecular basis for the observed interference with the virus' replication. Below, we discuss the major steps involved in the replication cycles of HSVs, as well as simple experimental approaches that can be used as route maps for identifying the mode of action of novel anti-HSV antivirals.

## Virion structure and composition

**Figure 2:**
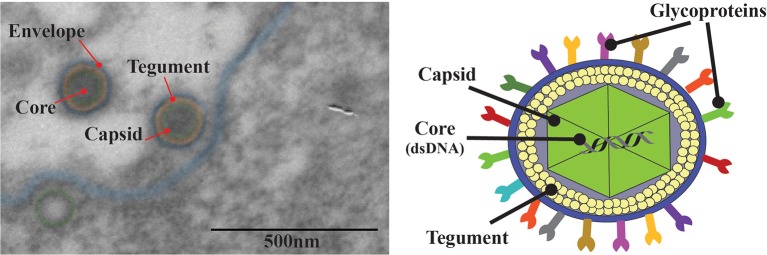
Structure of herpes simplex viruses. **Left:** Transmission electron microscopy (TEM) of HSV-2 infected Vero cells. The red arrow indicates the four main elements in the HSV-2 virion. An electron opaque core containing the viral DNA, an icosahedral capsid surrounding the capsid (green), the tegument that surrounds the capsid (orange) and an outer lipid bilayer envelope (blue). **Right:** Schematic illustration of HSV. The HSV virion is an enveloped, double-stranded DNA virus (~154 kpb) that encodes >80 genes; the viral genome is enclosed in an icosahedral capsid of ~125 nm surrounded by a complex mesh of viral proteins termed the tegument. HSV is enveloped with a lipid membrane that harbors numerous glycoproteins that protrude from the virion surface.

Like all members of the *Herpesviridae* family, HSV virions are composed of four main architectural features: envelope, tegument, capsid, and the viral genome (Pellet and Roizman, 2007) (Figure 2). Decades of study on HSV and novel techniques, such as cryo-electron microscopy (Dai and Zhou, 2018; Yuan et al., 2018) which provides < 5 Å resolution of the whole virion, have delivered valuable knowledge on the details of the structure and composition of these viruses (Grünewald et al., 2003; Brown and Newcomb, 2011). Electron microscopy analyses show that HSV virions have an icosahedral capsid with a diameter of ~125 nm contained within a spherical particle with an average diameter of 186 nm that extends up to 225 nm with the spikes of its numerous glycoproteins that protrude from the virus surface (Figure 2) (Grünewald et al., 2003; Brown and Newcomb, 2011; Lamers et al., 2015). The icosahedric capsid of these viruses encompasses linear, double-stranded viral DNA genomes that are ~150 kbp long, encode more than 80 genes and have high GC contents: 67 and 69% for HSV-1 and HSV-2, respectively (Kieff et al., 1971; Wu et al., 2016). Covering the capsid there is a collection of ~20 proteins defined as the tegument, which consists in proteins that have important roles in subverting the host antiviral response early after infection (Vittone et al., 2005; Loret et al., 2008; Kukhanova et al., 2014; Owen et al., 2015). Interestingly, recent electron cryo-microscopy analyses have evidenced interactions between capsid proteins such as U_L_17 and U_L_25, and tegument components, particularly VP1/2 which form the capsid vertex-specific component (CVSV) (Morgan et al., 1954). The tegument is in turn covered by a lipid envelope which harbors numerous viral glycoproteins from an assortment of at least 11-virally encoded glycoproteins (Figure 2) (Suazo et al., 2015a).

Virucides are antiviral compounds that can interfere directly with viral infectivity of the virion by altering their structure or components (Suazo et al., 2015b). Although their mechanism of action is seldom studied at the molecular level, some of these compounds interfere with the lipid envelope of HSVs, which can be evidenced by transmission electron microscopy (TEM) (Popkin et al., 2017). Interestingly, numerous compounds with virucidal activity have been identified both, derived from natural and non-natural sources (Schuhmacher et al., 2003; Schnitzler et al., 2007; Bultmann et al., 2010; Houston et al., 2017; Pradhan and Nguyen, 2018). Importantly, the identification of virucidal activity in an antiviral compound requires that the virus be treated alone with the drug for a defined period of time and then that the latter be washed before probing the infectivity of the virus over susceptible cells. Because virucides are generally disruptive in nature, they often have little specificity for particular family of viruses and furthermore may be lethal for the cell, thus their toxicity should be evaluated.

Besides analyzing the structure of HSV virions, evaluating their composition can also be key for identifying the mode of action of an antiviral. For this, the virus needs to be purified from infected cells, which is usually performed by passing cell supernatants from infected cultures through 0.45 μm filters, they are then ultracentrifuged at 20,000 g at 4°C. The composition of the sedimented virions can then be analyzed by western blot, by assessing the presence of structural viral proteins (Loret et al., 2008). At present, compositional analyses can be carried out on samples by mass spectrometry to precisely determine which viral proteins are present or absent in the viral particle (Engel et al., 2015; Kulej et al., 2017). Interestingly, this method can be performed on samples obtained from different cell compartments (prior cellular sub-fractioning), which provides viral proteomic profiles at different cellular locations (Bjornson et al., 2013). While this technique displays extraordinary resolution, sensitivity and depth of analysis, its implementation may be difficult and certainly requires costly equipment. Hence, western blot remains the most frequently used technique to assess protein composition of virions, although the results are less informative, as only few viral proteins are usually assessed at a time and protein quantification is not as precise as mass spectrometry.

## Herpes simplex virus binding to the host cell

**Figure 3:**
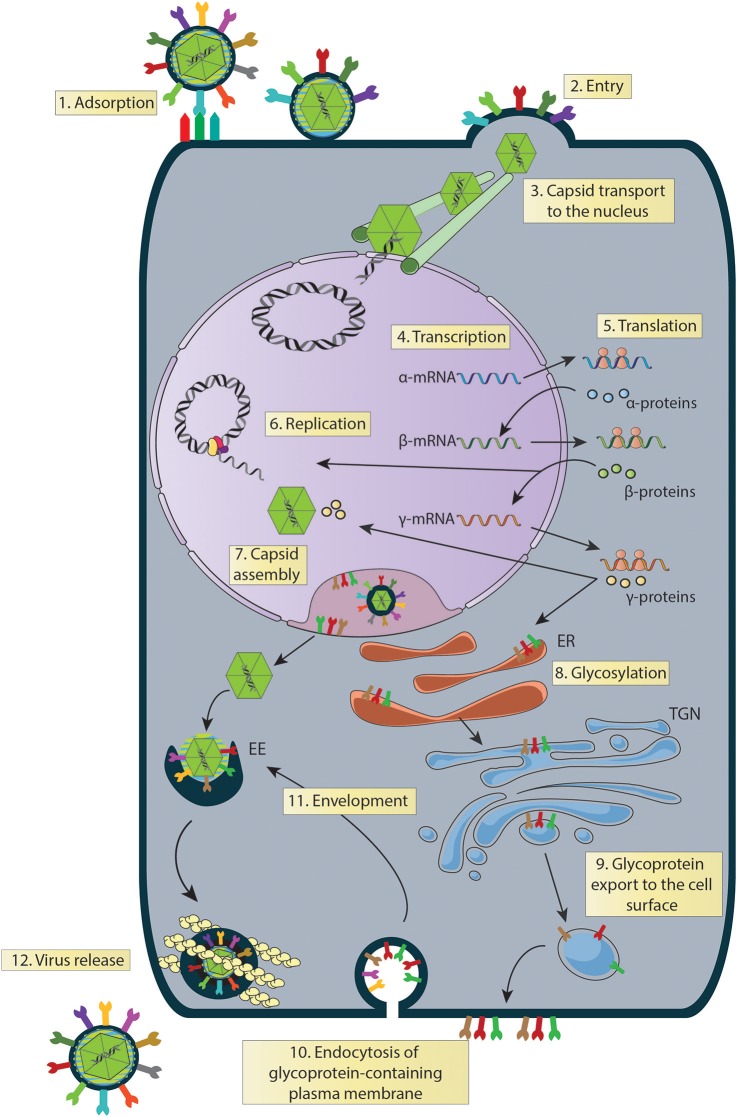
Steps of the HSV replication cycle that can be experimentally assessed to identify the mechanisms of action of anti-HSV drugs. (1) Binding: gB (HSV-1 and HSV-2) plus gC (HSV-1) participate in virus binding to the surface of the cells, which is followed by gD binding to one of its receptors. Virus binding blockade can be assessed at this stage by WB, FC, qPCR, among others. (2) Entry: after the fusion of membranes, the viral capsid and tegument proteins are internalized in the cytoplasm. Capsid internalization can be followed using a fluorescently-capsid-labeled HSV virus and assessed by LCM, and FC, among others. (3) Capsid transport to the nucleus: once in the cytoplasm, the viral capsid accumulates in the nucleus either by simple diffusion or aided by cytoskeletal structures, such as microtubules. Capsid accumulation at this site can be assessed by LCM. (4) Transcription: HSV genes are transcribed sequentially as α, β and γ genes. Transcripts can be detected by RT-qPCR at different time-points post-infection, respectively. (5) Translation: viral mRNAs are translated sequentially (α, β and γ proteins), which can be determined by WB. (6) Replication: viral genome replication occurs as a rolling circle. It can be assessed by qPCR. (7) Capsid Assembly: HSV capsids are assembled within the nucleus of infected cells and can be visualized by LCM, and TEM, among others. (8) Glycosylation: glycoproteins are translated and glycosylated in the endoplasmic reticulum (ER). Viral glycoprotein glycosylation can be assessed by WB. (9) Glycoprotein export to the cell surface: glycoproteins are processed in the trans-Golgi network (TGN) and multivesicular bodies (MVBs). Then, they are exported to the plasma membrane and can be followed by LCM using different markers such as TGN46, C6-NBD-cer, TGN38 for TGN and LAMP-1 for MVB. (10) Glycoprotein-containing plasma membrane endocytosis: endocytosis of glycoproteins can be followed by marking the cell surface with horseradish peroxidase (HRP) or by TEM. (11) Envelopment: glycoproteins within early endosomes (EE) fuse with capsids in the cytoplasm and can be followed/visualized using TEM, PALM, and STORM. (12) Virus release: virions in the extracellular medium can be determined by plaque assays. WB, Western blot; FC, flow cytometry; LCM, laser confocal microscopy; TEM, transmission electronic microscopy; PALM, photoactivated localization microscopy; STORM, stochastic optical reconstruction microscopy.

Infection of cells with herpes simplex viruses first requires that infectious virions bind to the cell surface using the viral glycoprotein B (gB) for HSV-1 and HSV-2 and additionally glycoprotein C (gC) for HSV-1 (Figure 3, Process 1 and Table 1) (Herold et al., 1991; Gerber et al., 1995; Atanasiu et al., 2010), although gC requirement for HSV-1 has been challenged (Rogalin and Heldwein, 2016). Though the main targets of gB and gC are heparan-sulfate proteoglycans (HSPGs), gB has also been reported to bind to the paired immunoglobulin-like type 2 receptor (PILR) on the cell surface (Satoh et al., 2008). Interestingly, binding of HSV to the cell surface has been reported to occur mainly at filopodia fibers, from which these viruses can surf until they reach the cell body (Oh et al., 2010). Noteworthy, several compounds acting as mimetics of HSPGs have been shown to possess antiviral properties by impairing viral adhesion to the cell surface by competitive inhibition (Gangji et al., 2018). Previous studies using synthetic carbohydrates, such us non-anticoagulant heparin, pentosan polysulfate and dextran sulfate, as well as natural compounds such as carrageenans and fucoidan obtained from sea algae, were shown to significantly reduce the infectivity of HSVs (Levendosky et al., 2015). On the other hand, inhibition of virus binding to the cell surface has been described for polyphenols contained within almond skin extracts, which were reported to bind to viral proteins (Bisignano et al., 2017), similar to the polyphenol Epigallocatechin gallate (EGCG), which was also reported to bind directly to HSV proteins (Colpitts and Schang, 2014). An antiviral effect was also reported for abalone hemocyanin, which specifically inhibited the adsorption of HSV to the cell surface by binding to gB, gC, and gD, likely by mimicking cellular receptors (Talaei Zanjani et al., 2016). Also comparative molecular modeling studies have suggested that lignin sulfates inhibit HSV replication in a molecular mimic way (Raghuraman et al., 2007).

**Table 1:** Experimental approaches for evaluating key steps in lytic HSV replication cycles.

**Step or process**	**Commonly used methodology (outline)**	**Limitations and advantages**	**Critical considerations**	**References**
Binding	Western blot targeting structural viral proteins on samples obtained from cells incubated with HSV at different MOI at low temperature. This assay allows determining the amount of surface-bound virus. Samples need to be incubated and processed under conditions that reduce the chances of virus internalization (low temperature). Cells need to be washed properly before protein extraction to remove excess unbound virus. Another experimental approach consists on the use of GFP-tagged virus. It can be done proceeding with infection at 4°C, washing and then performing confocal microscopy or flow cytometry.	Western blot is a routine technique in most laboratories. However, more qualitative than quantitative.	- Usually uses variable MOI to titer cell-surface bound virus (e.g., MOI 10, MOI 1, MOI 0.1). - Temperature: 4°C. - Incubation time: >1 h - Wash to remove unbound virus.	Atanasiu et al., 2010; Cheshenko et al., 2013; Ibáñez et al., 2017
Capsid entry into the cytoplasm	The number of internalized capsids is assessed using a recombinant virus that has a structural protein fused to a reporter (e.g., GFP). Infection is performed at low temperature. Cells are then washed and transferred at 37°C for 1–2 h. Finally, cells are trypsinized to remove any surface-bound virus and fixed prior to analysis. Virus-derived fluorescence can be measured by flow cytometry or by laser confocal microscopy.	Requires sophisticated equipment (flow cytometer or confocal microscope) and trained staff.	- High MOI (e.g., 100). - Structural reporter virus (e.g., HSV K26GFP) fluorescently labeled. - Temperature 4°C, then 37°C. - Wash, trypsinize cells to remove unbound and surface-bound virus. - Time: analyze at 1–2 hpi.	Desai and Person, 1998; Nicola and Straus, 2004; Wang et al., 2016; Ibáñez et al., 2017
Capsid transport to the nucleus	Cells are infected with a fluorescently-labeled virus (structural, e.g., K26GFP virus) at 37°C and viral capsids followed in live or fixed cells by laser confocal microscopy at 1–3 h post-infection. Membrane and nucleus stain inform about the relative distribution of the capsids within the cells. Alternatively, an approach using qPCR could be used (see text).	Requires access to confocal microscopy or TEM with trained staff. Cell fractioning (nucleus and cytoplasm) easy to perform. Western blot is a routine technique in most laboratories.	- High MOI 100–400. - Structural reporter virus (e.g., HSV K26GFP) fluorescently labeled. - Time: analyze at 1–3 hpi. - Stain cell membrane and nucleus for relative capsid position.	Desai and Person, 1998; Dohner et al., 2002; Petro et al., 2015
Viral gene transcription	Cells are infected at a MOI that infects 100% of cells. Extract RNA and analyze by RT-qPCR viral transcripts. Optimal time-point for analysis depends on the viral gene analyzed (α, β and γ genes) and may depend on the cell type infected.	Must choose correct timing. qPCR is nowadays a routine technique.	- MOI for 100% cell infection (>3). - Time: analyze gene expression at: 2–4 h for α genes 6–12 h for β genes 10–16 h for γ genes	Honess and Roizman, 1975; Roizman et al., 2013; Du et al., 2015
Viral gene translation	Western blot or flow cytometry analyses are performed on the infected cells to determine the expression of viral proteins. Optimal time-point for analysis depends on the viral gene analyzed (α, β, and γ genes) and may depend on the cell type infected.	Western blot is a routine technique in most laboratories. Flow cytometry requires equipment and trained staff.	- MOI for 100% cell infection (>3). - Time: analyze gene expression at: 2–4 h for α genes. 6–12 h for β genes. 10–16 h for γ genes.	Loret et al., 2008; Pasieka et al., 2008; Conway and Homa, 2011; Ma et al., 2017
Viral genome replication	Genome replication can be assessed by qPCR on total DNA extracted from infected cells at 8–24 h post-infection.	Must choose correct timing. qPCR considered a routine technique.	- MOI for 100% cell infection (>3). - Time: analyze 18–24 hpi.	Nystrom et al., 2004; Ibáñez et al., 2017
Capsid assembly	Transmission electron microscopy allows determining the presence and phenotypes of viral capsids (A and B without viral DNA and C with virus genome). Cells are infected and visualized at 6–8 h post-infection. Sucrose density gradients generated by ultracentrifugation can be performed when it is desired to detect capsid proteins by Western blot or assess the amount of viral DNA encapsidated by qPCR.	Sucrose density gradients requires ultracentrifuge. Western blot is a routine technique in most laboratories. qPCR considered a routine technique.	- MOI for 100% cell infection (>3). - Time: Analyze at 6–8 h.p.i. Multiple time-points are recommended.	Gibson and Roizman, 1972; Preston et al., 1983; Gao et al., 1994; McNab et al., 1998; Spencer et al., 1998; Dasgupta and Wilson, 1999; Taddeo et al., 2003; Turcotte et al., 2005; Sugimoto et al., 2008; Loret et al., 2012
Viral protein glycosylation	Western blot in viral proteins that undergo post-translational modifications. Gel-migration profiles are analyzed and can be compared with untreated cells.	Western blot is a routine technique in most laboratories.	- Viral glycoproteins (e.g., gB, gC, gD). - Time: 12 hpi.	Komuro et al., 1989; Pagano et al., 1989; Ladinsky and Howell, 1992; Futter et al., 1996; Turcotte et al., 2005; Calistri et al., 2007; Henaff et al., 2012
Glycoprotein export to the cell surface	Co-localization between TGN, MVB markers (with fluorescently-labeled antibodies) and viral glycoproteins can be determined by confocal laser microscopy 12 h.	Requires access to confocal microscopy with trained staff.	- Reported MOI 5. - Time: 12 hpi. - Organelle markers: TGN, MVB.	Avitabile et al., 2004; Calistri et al., 2007
Endocytosis of glycoproteins	Endocyted viral glycoproteins can be determined by electron microscopy by marking the cell surface with HRP and then localizing viral proteins with labeled antibodies. Cells are infected, labeled 12 h with HRP and then fixed. Antibody staining of viral glycoproteins can contribute determining the localization of these proteins.	Requires access to TEM with trained staff.	- Reported MOI 2. - Mark the cell Surface with HRP. - Antibodies against viral glycoproteins.	Foster et al., 2004; Hollinshead et al., 2012; Albecka et al., 2016
Capsid envelopment	Capsid envelopment can be assessed by confocal laser microscopy or transmission electron microscopy, using antibodies against host and virus proteins to determine the localization and assess whether they are located within early endosomes in the cytoplasm.	Requires access to confocal laser microscopy or transmission electron microscopy with trained staff.	- Reported MOI 2. - Time: analyzed at 12–24 hpi. - Organelle/Cell. compartment markers. Antibodies against viral glycoproteins.	Albecka et al., 2016
Virion release	Infective virus release can be assessed by plaque assays by performing serial dilutions of recovered supernatants, as well as lysed-cell preparations (to recover virus within the cells and unable to undergo exit) over HSV-susceptible cells. Sample collection can be done within 18–36 h post-infection.	Routine technique in most laboratories.	- Cell lysis strategy (must not damage the viral particles). - Time: analyze within 18–36 hpi.	Arens et al., 1988; Sugimoto et al., 2008; Fabiani et al., 2017

**Figure 4:**
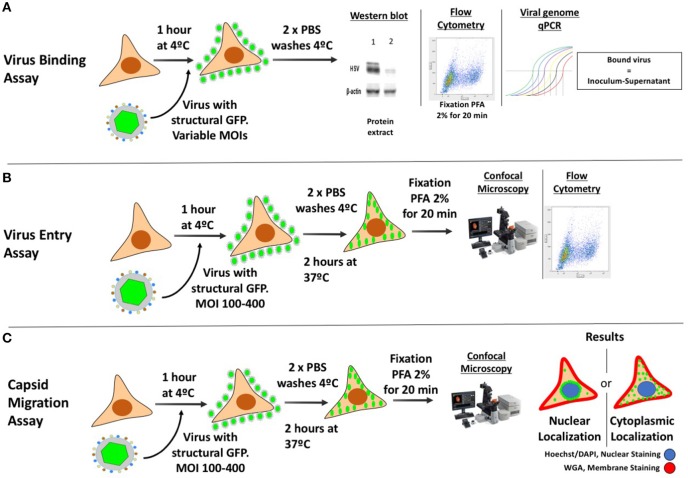
Methodologies for assessing binding, entry and viral capsid transport to the nucleus in HSV-infected cells. **(A)** Protocol for assessing the binding of HSV to the cell surface. The culture must be brought to 4°C and then inoculated with virus (with or without a structural reporter such as GFP) at different MOIs. The plate is incubated at 4°C for 4 h to allow the virus to adsorb to the cells without entering. Afterwards, 2 cold PBS washes are performed in order to wash the unbound virus and samples are then analyzed either by Western blot, blotting against viral structural proteins, by flow cytometry analyzing GFP (requires PFA fixation), or by qPCR quantifying viral genome (bound virus is equal to the difference between the inoculum titter and virus detected in the supernatant with unbound virus). **(B)** Protocol for assessing the entry of viral capsids into the cytoplasm. The culture is brought to 4°C and then inoculated with virus (with a structural reporter such as GFP). Afterwards, PBS washes will remove the unbound virus and the plate is then incubated at 37°C for 2 h to allow the coordinated entry of the viral capsids into the cell. Then, the cells are trypsinized, fixed with PFA and analyzed either by confocal microscopy or flow cytometry to determine the amount of GFP associated to the cells. **(C)** Protocol to assess viral capsid migration to the nucleus. The procedure is similar to that for assessing the entry of viral capsids into the cytoplasm, except that the sample are analyzed by confocal microscopy after staining the nucleus and cell membrane with dyes, such as DAPI and WGA, respectively.

A common strategy used to assess virus binding to the cell surface consists on quantifying, by western blot the amount of virions bound to the exterior of the cells after adding varying doses of HSV at 4°C (Figure 4A) (Cheshenko et al., 2013). Cells are infected with HSV at different multiplicities of infection (MOIs, 0.05–1) for several hours (usually 4 h) at 4°C to allow virus binding to the cell surface, while avoiding virus (capsid) internalization. After this incubation period, cells are washed with cold buffer and protein extractions are carried out directly over the cultures for performing western blots targeted at structural viral proteins, such as glycoproteins, tegument proteins or capsid proteins (Ibáñez et al., 2017). Different MOIs are used in this assay in order to titer the amount of virus bound to the cell surface, as saturating quantities of viral proteins in the blot could limit the quantification of potential differences between the analyzed groups (with and without antiviral drug) (Atanasiu et al., 2010; Cheshenko et al., 2013). Reduced virus binding to the cell surface can be visualized as less intense protein bands in the western blot at a particular MOI when compared to untreated cells. Hence, this assay indicates how much virus is bound to the cell surface with and without the drug. Alternatively, flow cytometry can be used to determine the amount of virions bound to the cell surface, by measuring the presence of viral proteins or a virus-encoded structural reporter (e.g., GFP) on the cell surface after virus-cell incubation at 4°C and fixation (Hadigal et al., 2015; Ibáñez et al., 2017) (Figure 4A). Similar to the assay described above, unbound viruses need to be washed out and low temperatures should be maintained throughout the experiment until fixation. Because the virion is intact, antibodies against virus-exposed epitopes should be used, such as surface glycoprotein antigens. As an alternative, the number of copies of viral genomes associated to the cell (bound) with in the supernatants (unbound) can be quantified by qPCR to assess whether differences occur over the adhesion of HSVs to the cell surface in the presence of a candidate antiviral drug and a known amount of virus added to the culture (Figure 4A) (Dai et al., 2018). Importantly, one must consider that differences in the amount of viral proteins detected could be given either by drug effects that act directly over viral glycoproteins, interference with virus-receptor interactions on the cell surface or by antiviral compounds that modulate the expression of virus receptors on the cell surface (Rogalin and Heldwein, 2016). The latter can be easily assessed by quantifying the expression of HSV receptors, such as nectin-1, nectin-2 and HVEM by flow cytometry on the cell surface (Akhtar et al., 2008).

## Herpes simplex virus entry into the host cell

Once the virus particles have adhered to the cell surface, the viral glycoprotein D (gD) will engage one of its target receptors, which depends on the cell type infected. While gD will preferentially bind to nectin-1 (HveC) or nectin-2 (HveB) in non-immune cells (Martinez and Spear, 2001; Richart et al., 2003), infection of immune cells is mainly thought to occur through gD-binding to HVEM (*Herpes Virus Entry Mediator, TNFRSF14*), a TNFR-receptor family member that interacts with numerous host ligands, both soluble and membrane-bound, and signals intracellularly depending on their specificity and orientation (*cis* vs. *trans*) (Whitbeck et al., 1997; Kovacs et al., 2009). Besides these receptors, additional gD ligands have been identified, such as 3-O-sulfated heparan sulfates which have been proposed to play relevant roles in HSV infections (Shukla et al., 1999). In this regard, viral entry can be blocked with antibodies that prevent the interaction between gD and its receptors (Criscuolo et al., 2018). For instance, M27f is a monoclonal antibody against gD that has shown neutralizing activity, not only against HSV-1 but also against HSV-2 and that inhibition of the entry process can occur even after attachment of virions to the cell surface (Du et al., 2017).

After binding to any of its ligands, gD will undergo conformational changes that activate the glycoprotein H and L (gH/gL) protein complex in the virus envelope (Atanasiu et al., 2010; Lazear et al., 2014). Once activated, the gH/gL glycoprotein complex will promote the activation of gB, which will act as the viral fusion protein that brings together the cell membrane and virus envelope, inducing the internalization of virus components into the cell cytoplasm (Figure 3, Process 2 and Table 1) (Atanasiu et al., 2010). While HSV entry occurs mainly through the fusion of the virus envelope with the plasma membrane in cell substrates for these viruses (Vero and HEp-2 cells: green monkey kidney cells and human epithelial cells, respectively) (Pellet and Roizman, 2007), HSV entry into other cells, such as CHO cells (Chinese Hamster Ovary cells) occurs mainly through a pH-dependent endocytosis route (Yuan et al., 2018). Noteworthy, virus entry via a phagocytosis-like uptake mechanism has also been reported (Clement et al., 2006). Interestingly, the gH/gL heterodimer has been shown to interact with cell surface integrins, particularly αvβ6, αvβ8, and αvβ3, with the first two capable of inducing the dissociation of the gH/gL heterodimer and independent activation of gH, which promotes HSV cell entry through acidic endosomes (Gianni et al., 2013, 2015; Cheshenko et al., 2014).

Because antiviral compounds could interfere with the HSV replication cycle at the step of virus entry into the cell, different strategies have been developed to assess this process. A common experimental methodology consists in detecting virus-derived fluorescence in the cytoplasm of infected cells by laser confocal microscopy or flow cytometry (Figure 4B). In this assay, a recombinant virus that has a structural protein fused to a reporter (e.g., green fluorescent protein, GFP) can be used. A frequently used virus for this purpose is an HSV-1 virus that has the capsid protein VP26 fused to GFP (HSV K26GFP) (Desai and Person, 1998). Because the GFP is fused to the viral capsid, following the GFP-derived fluorescence early after infection allows determining whether viral capsids have been internalized or not within the cells. Resolution will likely be improved if viruses bound to the exterior of the cells are eliminated. Using this virus, HSV entry can be assessed by incubating the virus and cells for 1 h at 4°C and then raising the temperature to 37° for 2 h, which will promote virus internalization (Ibáñez et al., 2017). After this period, cells are washed and treated with trypsin to remove non-internalized viruses and detach the cells that need then to be fixed (e.g., with paraformaldehyde, PFA), prior to analyses (Ibáñez et al., 2017). The fluorescence intensity derived from the GFP reporter will inform on the amount of virus that has been internalized into the cells, as well as the location of the capsid (see following section).

Alternatively, virus entry into target cells can be determined by transmission electron microscopy (TEM) by locating viral capsids within the cells at early time-points after infection. Although this technique allows direct visualization of viral structures within the cell, relatively few particles will likely be observed, which will depend on the MOI used (Nicola and Straus, 2004). Another methodology that can be used to evaluate viral entry is qPCR, by determining the amount of viral genomic DNA in infected cells at early time-points compared to adequate controls. In this assay, cells are incubated in ice to allow viral attachment and then incubated at 37°C for 30 min. Cells are washed, trypsinized and then DNA is extracted for performing qPCR (Wang et al., 2016).

## Capsid transport to the nucleus and genome delivery into this compartment

Once HSV particles have reached the cytoplasm (Figure 3, Process 2 and Table 1), numerous tegument proteins associated to the capsid will exert their functions in this space, while others such as the transactivator VP16 will migrate to the nucleus to promote the transcription of viral genes needed for the virus' replication cycle (Roizman and Zhou, 2015). Importantly, the viral capsids will also reach the nucleus to deliver the viral genetic material into this compartment to serve as a template for the transcription of viral genes, as well as viral genome replication (Figure 3, Process 3 and Table 1). Although simple diffusion of viral capsids within the cytoplasm has been suggested to be enough for their accumulation at the periphery of some cell types, other studies argue that capsid association with this compartment requires active transport mechanisms (reviewed in Garner, 2003). For instance, capsid localization in the nucleus has been related with microtubules and dynein-mediated transport in BHK and Vero cells, which was evidenced by the co-localization of capsids with these host proteins 1–2 h post-infection (determined by confocal microscopy). Consistently, microtubule-depolymerizing agents hampered capsid accumulation in the periphery of the nucleus (Sodeik et al., 1997). During this process, HSV capsids were reported to directly interact with host transport proteins dynein and kinesin-1, which enable anterograde and retrograde capsid movements through microtubules (Dohner et al., 2002; Radtke et al., 2010). The use of both, dynein and kinesin-1 by HSVs, which have opposing traveling capacities in polarized microtubules during the infection processes, could be explained by the fact that the microtubule-organizing centers may be distributed differently in distinct cell types. In this process, the viral tegument protein VP26 has been suggested to play a key role in the interaction between the capsids and dynein, by interacting with the dynein light chains RP3 and Tctex1, as determined in two-hybrid assays using a library of viral proteins tested for interactions with dynein subunits (Douglas et al., 2004). Consistent with this notion, naked capsids generated *in vitro* that lack VP26 are unable to mobilize to the cell nucleus, while capsids that express this protein do (Douglas et al., 2004). However, another study reported that an HSV-1 mutant lacking VP26 was able to reach the cell nucleus, similar to the wild-type HSV-1 suggesting that not only VP26 plays a role in capsid transport to the nucleus, but also other viral proteins and host receptors different from dynein (Döhner et al., 2006). Consistent with this observation, other studies have reported that a variety of inner tegument proteins can interact with either dynein or kinesin-1 and promote microtubule-mediated capsid transport to the nucleus (Abaitua et al., 2012). This is the case for the tegument proteins VP1/2 (also named U_L_36) and U_L_37, which have been reported to participate in capsid transport to the nucleus (Abaitua et al., 2012). A seven-residue deletion in the protein VP1/2 was shown to block the infection process and stall capsids at the microtubule organizing center, suggesting that the nuclear localization signal (NLS) in the VP1/2 protein is fundamental for the routing the viral genetic material to the nuclear pore complex (NPC) and docking at this site (Abaitua et al., 2012). A host factor described to participate in capsid transport to the nucleus is heat shock protein 90 (Hsp90), a chaperone (Zhong et al., 2014). Interestingly, pentagalloylglucose (PGG), a natural polyphenolic compound present in numerous medicinal herbs has been reported to delay the nuclear transport of viral particles to the nucleus, by decreasing the expression of dynein within the cell. Cells treated with PGG showed accumulated capsids in the cytoplasm when staining the capsid protein VP5 (Jin et al., 2016).

Related to the movements of capsids within infected cells, other reports suggest that HSV-1 engages dynamic microtubules (MTs) at early stages of infection, using plus end-tracking proteins (+TIPs) complexes composed of cytoplasmic linker protein 170 (CLIP-170), dynactin-1 (DCTN1) and end-binding protein (EB1), with retrograde capsid transport being dependent on CLIP-170 (Jovasevic et al., 2015). Interestingly, a recent report described that an interferon (IFN) response in primary rodent superior cervical ganglion (SCG) neurons reduced the axonal transport of capsids of *alphaherpesviruses* 24 h after infection, suggesting that some elements of the IFN responses use the same molecular motors than HSV capsids (Song et al., 2016).

One of the most common methodological approaches to evaluate the transport of capsids to the nucleus is based on confocal microscopy (Figure 4C). In order to follow the capsids within the cells, viruses such as the K26GFP virus encoding a structural GFP-fusion protein in the capsid and described above, can be used (Desai and Person, 1998). This recombinant virus permits localizing viral capsids in fixed or live cells during the infectious process (Desai and Person, 1998). Recently, we reported that treating cells with cobalt protoporphyrin (CoPP), a drug that induces the host factor heme oxigenase-1, has anti-HSV activity and interferes with the accumulation of K26GFP viral capsids around the nucleus, suggesting that its mode of action is interference with capsid transport to this compartment (Figure 4C) (Ibáñez et al., 2017). Another method for studying viral transport within infected cells, consists on determining the localization of capsids by transmission electronic microscopy. Since the magnifying power of this technique is greater than that of confocal microscopy, it is possible to visualize capsids as single particles within cells and precisely determine their location. Alternatively, viral capsids in TEM samples can also be marked with specific antibodies conjugated to electron-dense particles (e.g., gold particles) to visualize them (Dohner et al., 2002).

Once the capsids have migrated to the nucleus, the viral genome will be delivered into this compartment. This step is achieved by the docking of the capsid to nuclear pore complexes (NPCs) and the injection of the viral DNA into the cell nucleus (Batterson et al., 1983). Studies assessing host and viral proteins participating in the docking of capsids to the nuclear membrane have identified VP1/2 and nucleoporins Nup358 and Nup214 as a relevant factors that favor binding of capsids the nucleus of infected cells (Copeland et al., 2009). Other host and viral proteins participating in this process are integrin-β and viral protein U_L_25, which interact with nucleoporins CAN/Nup214 and hCG1 in order to dock to the capsids to NPCs. U_L_25 was also reported to interact with the capsid proteins VP1/2 and U_L_6 and to participate in triggering DNA release into the nucleus (Ojala et al., 2000; Pasdeloup et al., 2009).

The transfer of viral genomes into the nucleus can be determined by fluorescent *in situ* hybridization (FISH), by targeting viral genomic sequences early after infection (3 hpi) with a beacon (Everett and Murray, 2005). FISH directly assesses viral DNA translocation into the nucleus, which is uncoupled from the translocation of viral proteins such as ICP4, virion host shutoff protein (vhs) or VP16, which also migrate to this compartment early after infection (Everett and Murray, 2005; Cheshenko et al., 2014). On the other hand, to assess the viral DNA localization in the infected cells the viral DNA can be labeled with nucleoside analogs added to the cell culture during viral genome replication that are then marked for tracking the viral DNA (Wang et al., 2013; Sekine et al., 2017). Finally, a relatively simple approach for assessing whether viral genome delivery into this compartment has occurred is to perform qPCR of viral genomic DNA in nuclear extracts, obtained at early time points after infection, such as 1–3 h post-infection. Because viral DNA genome is being quantified, any viral gene encoded within the genome can be assessed. Importantly, such assays need to be controlled with similar qPCR assays performed in the cytoplasmic fraction, in such a way to control virus input and distribution within the infected cells. Controls for validating the purification of nuclear and cytoplasmic fractions can be performed by blotting, for example proteins that should be present only in the cytosol (e.g., GADPH) or nucleus (histones).

## Herpes simplex virus gene expression and viral genome replication

**Figure 5:**
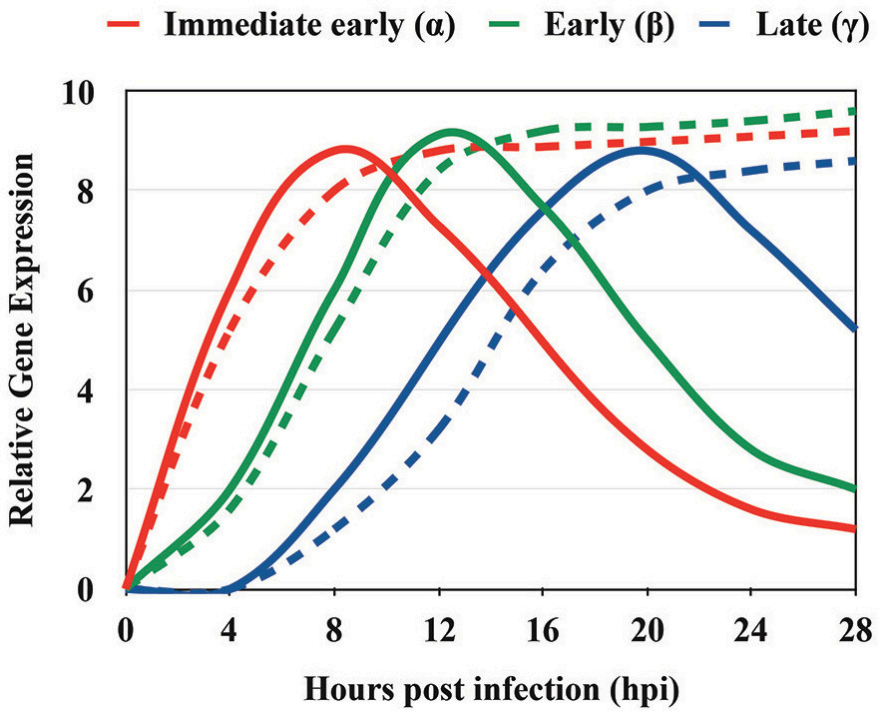
Approximate timing of gene expression of immediate early (α), early (β), and late (γ) HSV genes. Representative kinetic analysis of immediate early (red), early (green), and late (blue) HSV genes between 0 and 28 h post infection. The solid lines represent the characteristic expression pattern of HSV genes. The dashed lines indicate the expression of HSV genes that do not necessarily respond to the pattern of α, β, γ genes and overall continuously increase in time after infection.

Genomic HSV DNA is infectious *per se*, which means that infectious particles can be generated directly from cells transfected with purified viral DNA, without the need of any viral proteins accompanying this DNA; that is, immediate early viral genes (also named alpha genes) can be transcribed thanks to host-encoded factors (Honess and Roizman, 1974). Some of these immediate early-expressed viral proteins are: Infected Cell Protein (ICPs) 0, 4, 22, 27, and 47 (Honess and Roizman, 1975). Importantly, some of the proteins derived from these genes will in turn promote the expression of other viral genes that are controlled by these viral factors inducing two later successive waves of viral protein expression: one commanding the expression of early viral genes (also named beta genes) and a wave of late viral genes (or gamma genes) (Roizman and Zhou, 2015) (Figure 5). Hence, the virus will undergo sequential transcription of viral transcription factors such as for ICP0, among others (Kalamvoki and Roizman, 2008; Liang et al., 2009). Importantly, the method *Isolation of Proteins On Nascent DNA* (iPOND) has provided valuable information on the proteins both, cellular and viral that bind to HSV DNA and play roles in the transcription of viral genes and replication of the viral genome. In this technique, infected cells are incubated with a biotinylated nucleoside analog (5-ethynyl-2′-deoxyuridine, EdU) that labels viral DNA, which is then precipitated bringing down the proteins associated to this genetic material. This DNA is then analyzed by immunoblotting or mass spectrometry (Dembowski and DeLuca, 2015, 2018; Dungrawala and Cortez, 2015). Interestingly, this methodology has identified ICP4 and ICP8 as the most abundant viral proteins bound to the HSV genome, as well as several host chromatin-remodeling complexes bound to the viral DNA (Dembowski and DeLuca, 2015).

Immediate early genes reach peak rates of synthesis between 2 and 4 h after infection (Honess and Roizman, 1975; Roizman et al., 2013); early genes reach a peak of synthesis between 6 and 12 h post-infection (Roizman et al., 2013); and late genes reach peaks of synthesis between 10 and 16 h post-infection (Figure 3, Process 4 and Table 1) with the latter sometimes sub-divided into early-late (or gamma-1) and late (or gamma-2) genes (Figure 5 and Table 2). HSV genomes express more-less 84 transcripts that encode for proteins, as well as numerous long non-coding RNAs and 16–17 microRNAs (Roizman et al., 2013; Du et al., 2015). Transcription of viral genes within host cells can be easily performed by RT-qPCR (reverse-transcriptase PCR followed by quantitative PCR) of the viral genes. However, the expression of each type of viral genes will display a peak in transcription (abundance) at different time-points during the infection process. Although the timing for gene expression have been reported in the literature for each type of gene, optimal peak expression profiles for each type of gene frequently need to be determined empirically, as they may somewhat depend on the cell type infected and laboratory conditions. Although RT-qPCR measures RNA abundance, it does not measure transcription *per se*. In order to map the genome-wide distribution of transcriptionally engaged polymerase II at a base pair resolution in viral gene promoters, a *Precision nuclear Run-On* assay can be performed (PRO-seq). This approach uses biotin-labeled ribonucleotide triphosphate analogs (biotin-NTP) for nuclear run-on reactions, allowing affinity purification of nascent RNAs for high-throughput sequencing (Birkenheuer et al., 2018). Harmaline, a dihydro-pyrido-indole obtained from *Ophiorrhiza nicobarica*, which is an ethnomedicinal herb was found to inhibit HSV replication by interfering with the transcription of early viral genes, namely ICP0 by impairing the binding of an immediate-early complex to its promoter (Bag et al., 2014). Harmine, a beta-carbon alkaloid widely present in plants, has also been described to block the transcription of HSV-encoded genes, likely by inhibiting HSV-2-induced NF-κB activation, which is required for transcription of viral genes (Amici et al., 2006; Chen et al., 2015).

**Table 2:** Immediate early (α), early (β), and late (γ) HSV gene expression.

**HSV gene expression**	**Gene**	**Protein**	**Expression timing (hpi)**	**Cell line**	**References**
Immediate early (α)	*R_*L*_2*	ICP0	4–8	Vero, HeLa	Garvey et al., 2014; Lee et al., 2016
	*R_*S*_1*	ICP4	4–8	Vero	Garvey et al., 2014
	*U_*L*_54*	ICP27	4–8	Vero	Garvey et al., 2014
	*U_*S*_1*	ICP22	4–8	Vero	Garvey et al., 2014
	*U_*S*_12*	ICP47	4–8	Vero	Garvey et al., 2014
Early (β)	*U_*L*_23*	TK	8–12	Vero	Garvey et al., 2014
	*U_*L*_29*	ICP8	8–12	Vero	Garvey et al., 2014
	*U_*L*_50*	dUTPase	8–12	Vero, MRC5	Garvey et al., 2014; Fox et al., 2017
	*U_*L*_2*	Uracil Deoxyglycosylase	8–12	Vero	Garvey et al., 2014
Late (γ)	*U_*L*_48*	VP16	12–18	Vero, MRC5	Garvey et al., 2014; Fox et al., 2017
	*U_*L*_19*	VP5	12–18	Vero, MRC5	Garvey et al., 2014; Fox et al., 2017
	*U_*S*_6*	gD	12–18	Vero, MRC5	Garvey et al., 2014; Fox et al., 2017
	*U_*L*_27*	gB	12–18	Vero, MRC5	Garvey et al., 2014; Fox et al., 2017
	*U_*L*_53*	gK	12–18	Vero	Garvey et al., 2014
	*U_*L*_44*	gC	12–18	Vero, MRC5	Garvey et al., 2014
	*U_*L*_41*	*vhs*	12–18	Vero	Garvey et al., 2014; Fox et al., 2017

Furthermore, the expression of viral mRNAs can be followed by viral protein expression, which can be assessed by western blot and flow cytometry (Loret et al., 2008; Ma et al., 2017). While immediate early genes are mainly dedicated at blocking the cellular antiviral host response, such as interferon pathways, early genes are mostly focused in supporting the replication of the viral genome (Pasieka et al., 2008). On the other hand, late genes mostly encode for structural viral proteins that will be included in the infectious viral particle that will egress from the infected cell (Figure 3, Process 5 and Table 1) (Conway and Homa, 2011). An alternative approach to assessing viral protein expression is utilizing recombinant viruses that encode reporter genes. A practical tool for studying the transcription and translation of viral genes within the infectious cycle of HSV consists again on the use of viruses such as K26GFP HSV-1, although these experiments need to be performed at a low MOI in order to avoid background GFP fluorescence derived from input virus (Desai and Person, 1998). Other recombinant viruses that encode reporter genes under the control of immediate early (alpha) (Cun et al., 2006), early (beta) (Potel et al., 2002), and late (gamma) (Loomis et al., 2003; Harper et al., 2010) viral gene promoters are also of great utility for this purpose.

HSV genomes are circularized within the nucleus of the infected cells for replication to occur. The viral genome will be copied in these cells as rolling circles that produce concatemers, consisting on several copies of the entire viral genome in a linear form (Skaliter and Lehman, 1994). Determination of HSV genome replication can be assessed experimentally by performing qPCRs kinetics for DNA sequences encoded within the viral genome. Because replication of the genome is of interest in these assays, rather than defining particular locations, such qPCRs can be performed over total DNA extracted from the infected cells at time-points usually within 6–12 h post-infection to evidence an increase in viral genome copies (Figure 3, Process 6 and Table 1) (Nystrom et al., 2004; Ibáñez et al., 2017). Alternatively, the iPOND technique described above can also be utilized to quantify viral genome replication by staining the biotinylated EdU-labeled nascent viral DNA with a fluorescently-labeled molecule (e.g., an antibody against biotin or streptavidin labeled with a fluorophore) and quantifying fluorescence intensity in the cell nucleus by confocal microscopy (Reyes et al., 2017).

Overall, these assays allow determining the transcription and replication of the HSV genetic material, and are rapid and easy to perform in any laboratory with conventional equipment.

## Viral capsid assembly in the nucleus and transport to the cytosol

Following the transcription and translation of viral genes that are involved in viral genome replication (mainly early viral genes), expression of late viral genes mostly encoding virus structural proteins is initiated. A characteristic of HSVs is that capsids are assembled within the nucleus of infected cells (Morgan et al., 1954). Viral capsid proteins that are synthetized in the cytoplasm are translocated to the nucleus thanks to nuclear localization sequences (NLS) that signal these proteins to this compartment where viral genome replication takes place (Figure 3, process 7 and Table 1) (Abaitua et al., 2012; Sankhala et al., 2016). HSV capsids are mainly composed of the major capsid protein VP5 and also by other less abundant viral proteins, such as VP19C, VP23, and VP26 (Thomsen et al., 1994; Homa and Brown, 1997; Kobayashi et al., 2017). In addition to viral capsid proteins, assembly of this structure in the nucleus requires the presence and participation of an HSV-encoded protease and scaffolding protein named pre-VP22a, which cooperates in the formation of a viral capsid that can harbor the viral genome in its interior (Nicholson et al., 1994; Newcomb et al., 2001). Capsids will first be assembled as empty cages without any viral DNA, which will then later be packaged within this structure with the help of at least seven viral HSV-encoded proteins, namely U_L_6, U_L_15, U_L_17, U_L_25, U_L_28, U_L_32, and U_L_33 (al-Kobaisi et al., 1991; Patel et al., 1996; Baines et al., 1997; Lamberti and Weller, 1998; Salmon and Baines, 1998; Taus and Baines, 1998; Taus et al., 1998). Together, these components will assemble pro-capsids, which are somewhat thermodynamically unstable (Heming et al., 2017). Nevertheless, at least three different types of capsids have been thoroughly described within infected cells, which mainly differ on the viral material present within the cavity. While type A and type B capsids do not have viral DNA, type C capsids contain the virus genome and therefore are those that will ultimately produce infectious viral particles (McNab et al., 1998). Isolation of these three types of capsids can be performed using sucrose density gradients generated by ultracentrifugation (Gibson and Roizman, 1972). Furthermore, electron microscopy and more recently cryo-electron microscopy can also be used to study the presence and phenotype of viral capsids generated 18-24 h post-infection with HSV-1 (Homa and Brown, 1997; Heming et al., 2017). Additionally, a modified cryo-electron microscopy technique, based on the detection of gas bubbles generated due to radiation-induced damage in the sample has allowed localizing internal capsid proteins within HSV-1 (Newcomb et al., 1999; Wu et al., 2016). Besides capsid visualization, capsid composition is also informative at this stage, which can be determined with western blots to evaluate the presence and quantities of viral proteins such as VP5, VP23, VP26, U_L_17, and U_L_25, which should be present in these structures (Spencer et al., 1998; Dasgupta and Wilson, 1999). For this, nuclei of infected cells are separated from the cytoplasm and then fractionated so that capsids can be analyzed separately from host protein complexes. This isolation process is frequently done by disrupting the cells with NP40-based lysis buffers and subsequently ultracentrifugation in sucrose gradients (Taddeo et al., 2003). Interestingly, an antiviral effect for 5-chloro-1,3 dihydroxyacridone, an acridone derivative was reported at the stage of virus assembly and maturation resulting in reduced levels of encapsidated DNA. This was evidenced using TEM for capsid morphology analysis and rate-zonal ultracentrifugation for determining the protein composition of the resulting capsids (Akanitapichat and Bastow, 2002). On the other hand, WAY-150138, a thiourea compound was found to inhibit the HSV replication cycle by preventing DNA encapsidation, which was similarly evidenced by TEM and western blot analyses to determine the composition of these capsids. Interestingly, drug treatment depleted the viral proteins U_L_6 and U_L_15 from the capsids, which are involved in DNA entry and packaging (Newcomb and Brown, 2002).

Interestingly, a study reported that the three main types of HSV nuclear capsids described above can also be determined by flow cytometry. This approach stains viral DNA with Syto13, a fluorescent nucleic-acid stain (Loret et al., 2012). Based on different light-scattering properties, three regions of interest with different capsid populations can be arbitrarily defined: Region (i) consisting of A-capsids that do not display any fluorescent signal at all; Region (ii) consisting of B-capsids that display an intermediate fluorescence signal and Region (iii) corresponding to C-capsids which have the highest fluorescence signal. These regions can be separated by slope-change criterions in the SSC channel, determining the boundary between two fractions. Furthermore, this approach showed to be consistent with gold standard techniques used to analyse capsids, such as electron microscopy and DNA detection within viral capsids by qPCR through the amplification of *U*_*L*_*20* (Loret et al., 2012). Equipment designed for assessing the properties of small particles, namely exosomes, should likely provide size properties of HSV particles and contribute to the assessment of potential alterations in the overall structure of capsids (Liga et al., 2015).

**Figure 6:**
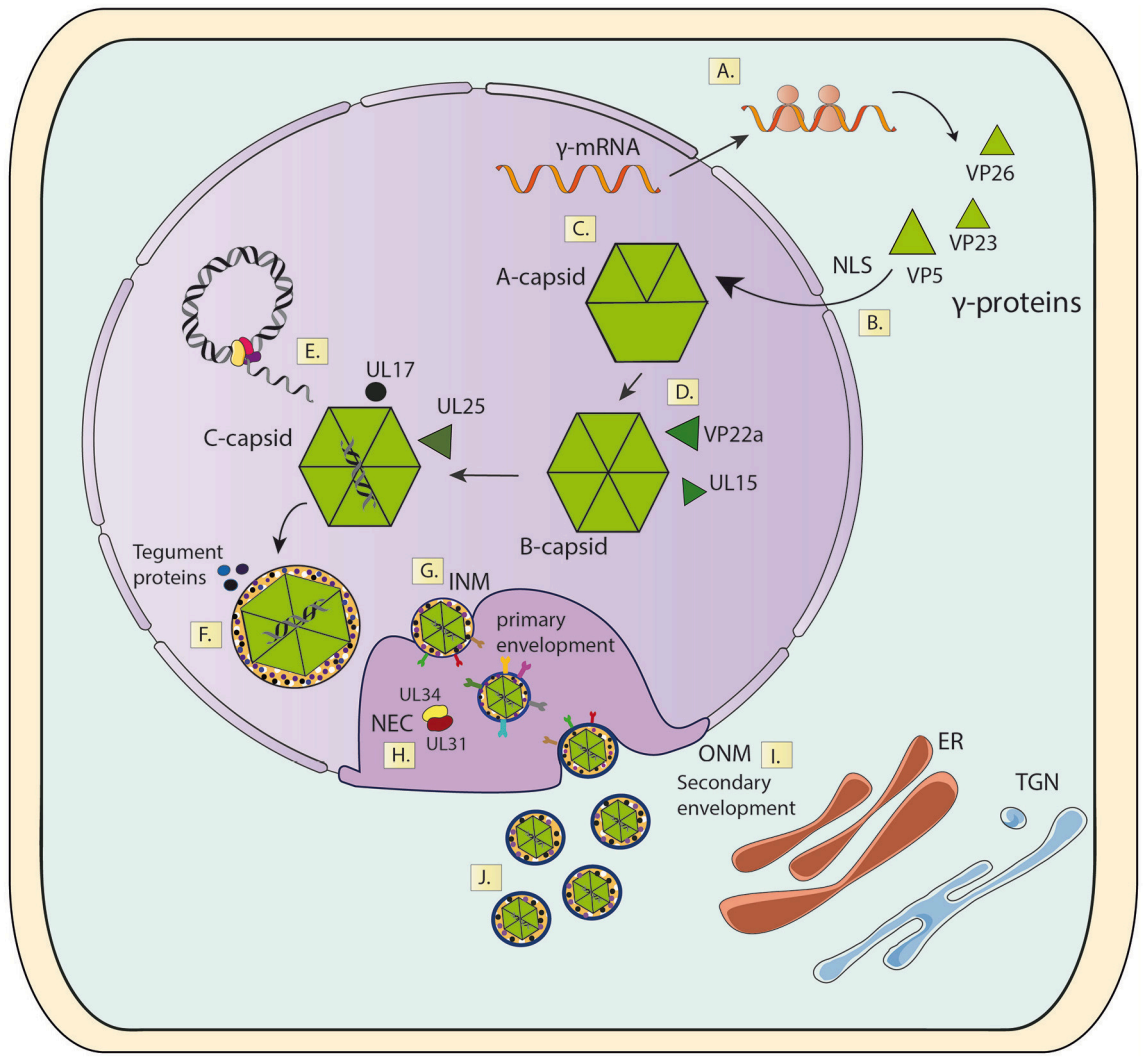
Viral capsid assembly in the nucleus and transport to the cytosol. **(A)** γ-mRNA translation. **(B)** Import of capsid proteins into the nucleus. **(C)** Capsid Assembly. **(D)** Capsid Maturation. **(E)** Viral genome packaging. **(F)** Initial tegument assembly. **(G)** Capsid envelopment in the INM. **(H)** Capsid envelopment in the perinuclear space. **(I)** Fusion of enveloped nuclear capsids with the ONM. **(J)** Release of mature capsids into the cytoplasm. INM, Inner Nuclear Membrane; ONM, Outer Nuclear Membrane; NEC, Nuclear Export Complex; NLS, Nuclear Localization Sequences; ER, Endoplasmic Reticulum; TG, Trans-Golgi Network.

Once the assembly of viral capsids has been completed in the nucleus, these particles will continue their maturation process in this same compartment through the acquisition of tegument proteins (Figure 6). Indeed, tegument proteins such as VP1/2 and U_L_17 will be added to the capsid at this stage, allowing the particles to leave the nucleus through the nuclear envelope (Bucks et al., 2007; Owen et al., 2015). The movement of viral capsids out of the nucleus is associated with nuclear actin filaments, as demonstrated by using depolymerization inhibitors of actin and myosin V (Forest et al., 2005). Because the diameter of the viral capsids is ~120 nm wide and the nuclear pores only support the passage of particles that are up to 36 nm in diameter, HSV capsids exit the nucleus by traversing the different nuclear membranes and space in between, namely the inner nuclear membrane (INM), perinuclear lumen and the outer nuclear membrane (ONM) by envelopment and fusion processes (Mettenleiter et al., 2013). First, the capsids acquire a primary envelope in the INM, which leads to the formation of primary-enveloped virions present in the perinuclear space (Mettenleiter et al., 2013). Then, a nuclear egress complex (NEC) composed of two viral proteins is required for egressing from this compartment. The first protein consists in the type-II viral membrane U_L_34 protein, which is anchored to the nuclear envelope and the second is the soluble viral protein U_L_31 (Ott et al., 2011). In the absence of this NEC, the nuclear egress of viral particles is blocked and capsids accumulate in the nucleus (Reynolds et al., 2002). An HSV with a mutation in the N-terminal domain of U_L_31 displays hampered translocation of capsids from the nucleus to the cytoplasm, which was linked with deficiencies in primary envelopment (Roller et al., 2010; Funk et al., 2015). Interestingly, the Hsp90 protein inhibitor AT-533 has been shown to block viral nuclear egress by inhibiting the functions U_L_31 and U_L_34 in cells infected with HSV-1 (Li et al., 2018). On the other hand, NEC also recruits viral kinases U_S_3 and U_L_13, as well as cellular protein kinases, such as PKC that phosphorylate and locally disrupt the nuclear lamina which facilitates capsid access to the INM by an envelopment process (Bjerke and Roller, 2006; Cano-Monreal et al., 2009; Wild et al., 2015). After reaching the INM, C-capsids are specifically selected to egress the nucleus over immature forms of the capsid (A- or B-capsids), likely thanks to the presence of the viral proteins U_L_17 and U_L_25, which are associated the mature form of C-capsids (Taus et al., 1998; Thurlow et al., 2006; Cockrell et al., 2011). Once in the perinuclear space, enveloped capsids will fuse with the ONM releasing capsids with some tegument proteins into the cytosol for further maturation (Granzow et al., 2001; Skepper et al., 2001; Panté and Kann, 2002). Numerous viral proteins participate in this process, such as viral glycoproteins, U_S_3 and U_L_31, among others (Figure 6) (Mou et al., 2009; Mettenleiter et al., 2013).

One way to analyze HSV particles that leave the nucleus of infected cells is to synchronize the infectious cycle within this compartment as a starting point. This can be achieved thanks to specific mutations in viral proteins that stall the virus at specific replication stages within the cells. An example is an HSV mutant that encodes a thermosensitive U_L_26 protease, which is required for capsid maturation and DNA encapsidation in the nucleus (Preston et al., 1983; Gao et al., 1994; Turcotte et al., 2005). This mutant suffers capsid accumulation in the nucleus when infected cells are incubated at 39°C. However, if cells are transferred to a permissive temperature (31°C) the capsids will proceed synchronously with the maturation process that follows the action of the U_L_26 protease and continue the exit process (Preston et al., 1983; Gao et al., 1994). Taken together, this procedure allows the accumulation of a significant amount of capsids within the nucleus and simultaneously synchronizes them for initiating their maturation process together, which can then be followed throughout different cell compartments. Importantly, this method will allow overcoming the limitations of having low numbers of virions per compartment under normal infection and the dispersion of virions in different subcellular compartments with different maturation levels (Sugimoto et al., 2008). This approximation was used to study the antiviral effect of brefeldin A (BFA) on HSV assembly. Cells were infected with the virus mutant and maintained at 39°C to induce the reversible accumulation of a population of procapsids. Then, cells were treated with BFA and shifted to 31°C for 3 h in order to let the accumulated procapsids mature. Results obtained using electron microscopy revealed an inhibition in the budding of capsids from the INM, because procapsids matured and packaged the viral genome, yet remained non- enveloped in this space and failed to exit the nucleus (Dasgupta and Wilson, 2001).

## Virion maturation in the cytoplasm

After leaving the nucleus (Figure 3 step 11), additional tegument proteins will be added to the capsids in the cytoplasm, such as U_L_7, U_L_11, U_L_16, and U_L_51 (Owen et al., 2015). Several tegument proteins have been described to partially localize at both compartments, such as VP1/2, U_L_37, U_L_41 (vhs), U_L_46, U_L_47 (VP13/14), U_L_48 (VP16), U_L_49 (VP22), although it is not clear whether these proteins are associated with capsids inside the nucleus or not (Pomeranz and Blaho, 1999; Donnelly and Elliott, 2001; Bucks et al., 2007). Due to the diversity of components that conform the tegument, it has been hypothesized that a subset of internal tegument proteins, including VP1/2 will anchor to the capsid and act as a scaffold protein to allow other tegument proteins to build upon it through the interaction with capsid proteins such as U_L_19 (VP5), U_L_17, and U_L_25 (Newcomb and Brown, 2010). A screen using mutant HSV viruses identified U_L_25 and VP1/2 as essential determinants for the binding of tegument proteins to the capsids (Coller et al., 2007). Finally, VP1/2 has been shown to interact with the tegument protein U_L_37, which further supports the bridging of tegument proteins with the viral capsids (Kelly et al., 2012).

Given that viral capsid assembly and their maturation involve several viral proteins and numerous cell compartments that make analyzing alterations in these processes complex, a frequently-used approach to determine interference with these steps consists on using transmission electron microscopy. TEM allows determining where viral particles accumulate, by evidencing a collection of capsids at specific compartments within infected cells, such as the nucleus, perinuclear space or cytoplasm, among others (Kaufman, 1962; Nicola and Straus, 2004; Cheshenko et al., 2014; Roizman and Zhou, 2015; Wang et al., 2016). Importantly, reporter proteins fused to viral determinants, such as GFP will again help following the migration and accumulation of relevant viral components throughout the cell thanks to confocal microscopy (reviewed in Hogue et al., 2015). The recent development of a triple fluorescent-tagged HSV that has the capsid protein VP26, tegument protein VP22 and envelope protein gB fused to yellow-, red-, and cyan-fluorescent proteins, respectively allows the simultaneous tracking of all three viral components in living cells by confocal microscopy (Sugimoto et al., 2008). The use of this triple-fluorescent-labeled HSV particles has contributed in identifying in which cellular compartment do viral components accumulate after drug treatments (Sugimoto et al., 2008). Also, cellular markers such as endoplasmic reticulum and Golgi apparatus molecular markers TGN46 for TGN, GM-130 for cis-Golgi, Golgi 58K for cis-, medial-, and trans-Golgi and EEA1 for endosomes can be used in order to visualize the specific compartments in which viral components accumulate (reviewed in Henaff et al., 2012). Such a virus can thus be used to determine the mode of action of specific drugs that are suspected to interfere with the maturation of viral capsids and virion assembly in the cell before their release into the extracellular medium (Sugimoto et al., 2008).

Despite the enormous potential of recombinant viruses that encode reporter genes as tools for identifying mechanisms of action of drugs, electron microscopy remains somewhat the most-frequently used technique to study virion maturation and exit, because of the ease of this approach in identifying at which compartment viral particles are trapped. Yet, with appropriate molecular markers for differentiating distinct compartments, it is likely that confocal microscopy will be as good or better than TEM for this analysis. Most likely the combination of both assays, first TEM and then confocal microscopy would help focusing analyses on particular cellular compartments.

Alternatively, cellular sub-fractioning and analyses of fractions by western blot against specific viral proteins may also be informative to localize viral structures at different compartments and hence, narrow down the mode of action of novel antiviral drugs.

## Virion maturation and release into the extracellular medium

While viral capsids are assembled in the nucleus and released into the cytoplasm, HSV glycoproteins are translated into the endoplasmic reticulum from which they will be derived into the *trans* Golgi network (TGN) (Wisner and Johnson, 2004; Turcotte et al., 2005) and then directed to multi-vesicular bodies (MVBs) (Figure 3, process 8 and Table 1) (Calistri et al., 2007). After the viral glycoproteins have reached the TGN and MVBs, these proteins will be exported to the plasma membrane (Figure 3, Process 9) (Alconada et al., 1999; Brideau et al., 2000; Beitia Ortiz de Zarate et al., 2004). Once on the cell surface, these proteins will be endocytosed and returned to early endosomes (Figure 3, Process 10 and Table 1) (Alconada et al., 1999; Hollinshead et al., 2012). Viral capsids in the cytoplasm will then fuse with HSV-glycoprotein-containing endosomes to form infectious virions within vesicles (Figure 3, Process 11 and Table 1) (Albecka et al., 2016). Finally, the virions within these vesicles will be secreted into the extracellular medium after traversing the actin mesh in the inner face of the plasma membrane (Figure 3, Process 12 and Table 1) (Hollinshead et al., 2012).

Because the above-mentioned processes involved in the replication cycles of HSVs depend on host-vesicle transport, the antiviral effect of ABMA, [1-adamantyl (5-bromo-2-methoxybenzyl) amine], a small molecule that acts selectively on host-endosomal trafficking was investigated in HSV-2 infection. Interestingly, ABMA was shown to inhibit early events in the infection process of HSV-2, but also later stages when applied 6–18 h post-infection, corresponding to the final steps of the replication cycle of HSVs. Importantly, ABMA was shown to confer significant protection against intravaginal challenge with HSV-2 in female BALB/c mice by reducing virus loads, among others (Dai et al., 2018).

**Figure 7:**
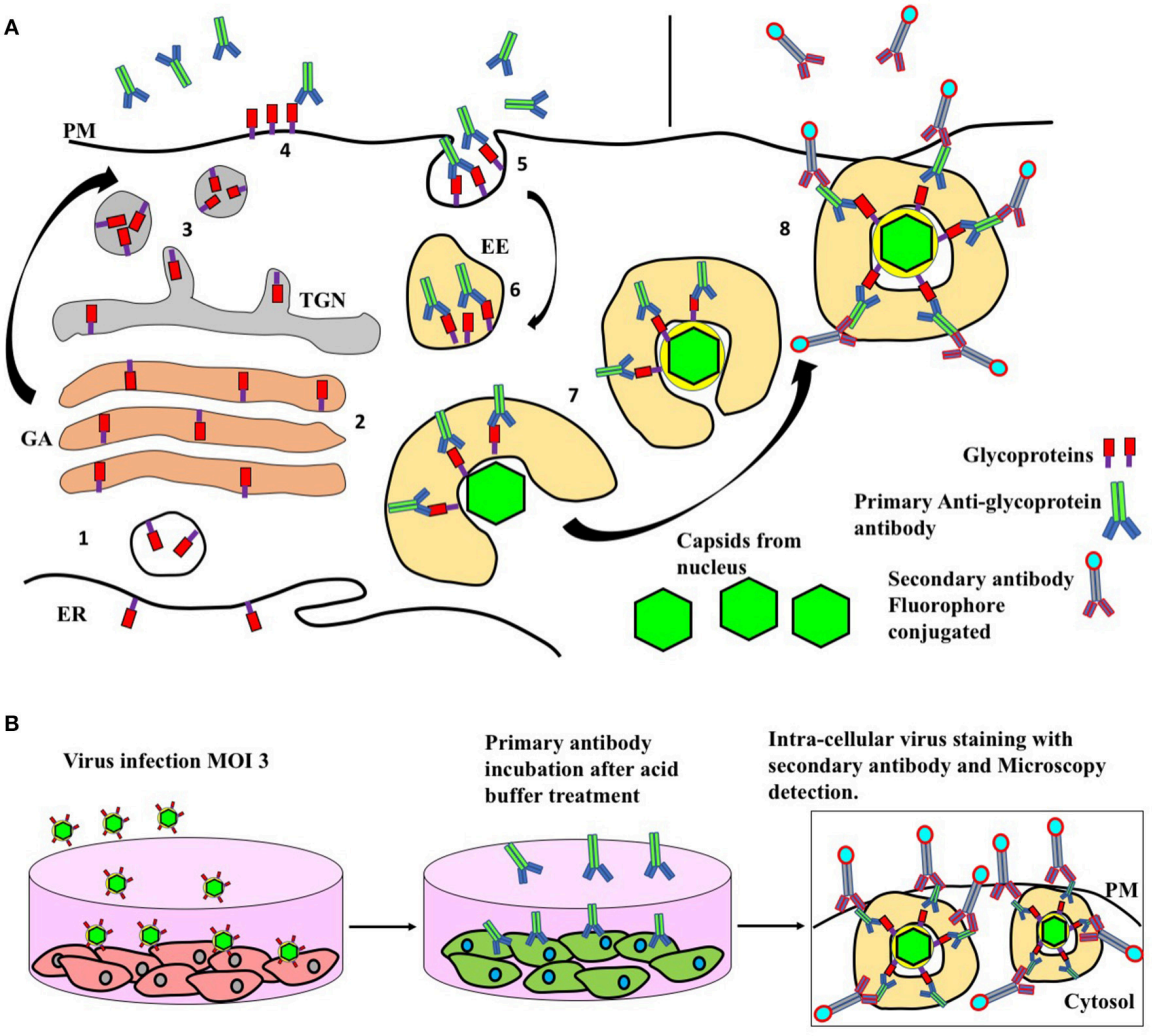
Endocytic pathway as a source of HSV envelope and experimental evaluation. **(A)** 1. Synthesis of viral glycoproteins in the endoplasmic reticulum. 2. Post-translational modifications of viral glycoproteins through Golgi apparatus. 3 Glycoproteins arrived at trans Golgi network and transport to the plasma membrane. 4. Glycoproteins arrive at the plasma membrane exposed to the extracellular space. 5. Staining of viral glycoproteins with primary antibodies and endocytosis. 6. Transport of antibody-stained viral glycoproteins through early endosomes. 7. Antibody-stained glycoprotein-containing tubules wrap cytoplasmic capsids forming virions with a double membrane. Cell fixing and secondary antibody fluorophore-conjugated staining for confocal microscopy evaluation. ER, endoplasmic reticulum; GA, Golgi apparatus; PM, plasma membrane; TGN, trans Golgi network; EE, early endosomes. **(B)** In order to study the process of virion maturation in the cytoplasm, it is possible to perform an antibody uptake assay. First, a confluent plate of cells is infected, 1 h after the cells are washed with acid buffer in order to inactivate residual viral particles. Then the cells are washed with PBS and incubated in free bovine serum medium with anti-glycoprotein primary antibodies for 16 h. Then, the cells are fixed and stained with a secondary antibody conjugated with a dye for immunofluorescence detection.

To determine the localization of viral glycoproteins within the TGN, confocal laser microscopy could be carried out using Giantin as a molecular marker and fluorescently-labeled antibodies against HSV glycoproteins (Figure 3, Process 9 and Table 1) (Henaff et al., 2012). Other TGN and HSV markers can also be used, such as TGN46, C6-NBD-cer, Golgin 97, and TGN38, as well as the viral protein ICP0 (Komuro et al., 1989; Pagano et al., 1989; Ladinsky and Howell, 1992; Turcotte et al., 2005). To assess whether MVBs also contain these viral components, the LAMP-1 marker can be used together with a marker for the viral glycoprotein B (Futter et al., 1996; Turcotte et al., 2005; Calistri et al., 2007). While LAMP-1 displays a dispersed pattern throughout the cytoplasm in uninfected cells, cells infected with HSV (12–24 h post-infection) show the LAMP-1 marker concentrated in MVBs and late endosomes (Calistri et al., 2007). Alternatively, the endocytosis process of viral glycoproteins from the cell surface has been assessed by labeling the outer membrane of the cell with horseradish peroxidase (HRP) and then localizing the activity of this reporter within different cell compartments by electron microscopy, while co-staining against viral glycoproteins (Hollinshead et al., 2012) (Figure 7). A similar approach can be performed by laser confocal microscopy, by labeling proteins of interest both, from the host and virus (Figure 3, Process 10 and Table 1) (Foster et al., 2004; Albecka et al., 2016). Furthermore, other types of microscopies that have been recently developed, such as photoactivated localization microscopy (PALM) and stochastic optical reconstruction microscopy (STORM) could be used to closely follow virion egress from infected cells (Fernández-Suárez and Ting, 2008; Hogue et al., 2014, 2016; Laine et al., 2015). Remarkably, these new microscopy techniques have allowed observing viral component at subcellular localizations within living cells infected with HSV at high resolutions (Figure 3, Processes 8, 9, 10, 11).

On the other hand, analyzing the glycosylation levels of HSV glycoproteins at this stage could also be informative. This post-translational modification could be determined by performing western blots against viral glycoproteins and comparing the migration profiles of these proteins relative to that of untreated cells (Johnson and Spear, 1983). For instance, gB usually displays an apparent electrophoretic weight of 115 and 100 kDa, which could vary upon treatment with a drug that interferes with its glycosylation pattern (Avitabile et al., 2004; Calistri et al., 2007).

Virion exit from infected cells involves their passing through a physical barrier in the inner plasma membrane side which consists on a curtain of F-actin (Schumacher et al., 2005; Roberts and Baines, 2010). To surpass this structure, HSV has been reported to activate myosin Va (myoVa), an actin motor, which through ATP hydrolysis is capable of mobilizing cargo along polar actin fibers toward its plus end (Roberts and Baines, 2010). Consistent with this finding, cells transformed with a myoVa-negative dominant were found to secrete less virus and retained more viral structures than infected control cells; Virus trapped within infected cells was released by freezing and thawing the cells (Roberts and Baines, 2010). Given these findings, it is possible to conceive scenarios in which antivirals may block the final steps of the infectious cycle and interfere with virus release into the extracellular medium. Evaluation of interference with the HSV replication cycle at this step could be assessed by lysing infected cells and analyzing whether the released content has infectious viral particles that were trapped within the cells and unable to undergo the final exit steps. Two common strategies for lysing cells and recovering virus particles within them for posterior tittering are freeze-thaw processes, as described above and sonication of cells to interrupt the cell membrane and release the cytoplasmic content (Arens et al., 1988). The content of the lysed cells can then be evaluated over HSV-susceptible cells, such as Vero cells for determining the presence of infectious particles within treated cells, as determined by counting plaque forming units (Figure 3, Process 12 and Table 1).

A high-throughput method for titrating HSV-1 uses a laser-based scanning in near-infrared with fluorophore-conjugated antibodies. Serial dilutions of virus are performed over monolayers of HSV-susceptible cells and then plaques counted thanks to a NIR-conjugated antibody against gB. Scanning results in fluorescence intensity values are then interpolated into a standard curve which converts the date into plaque forming units. This method has been validated for antiviral drug screening using acyclovir as a control (Fabiani et al., 2017).

Finally, an important factor to consider when dissecting the HSV replication cycle is the destination of virions, which may depend on the type of cell infected. While most cells infected with HSV will release abundant amounts of virions into the supernatant, a fraction of virions within the cell may be directed to cell-cell contact areas depending on the cell infected. For instance, in epithelial cells, virions are mainly transported to cell junctions in the plasma membrane (Wisner and Johnson, 2004). Transport selectivity to cell junctions has been suggested to be mediated by the viral gE/gI complex in epithelial cells and in combination with U_S_9 in neurons (Snyder et al., 2008). One model suggests that the cytoplasmic domains of the proteins from this complex extend from the vesicles that contain the virions and tether these proteins onto kinesin motors (Kramer and Enquist, 2013). Consistent with this model, mutations in gE, gI or in the cytoplasmic domain of gE reduce the numbers of HSV particles at cell-cell junctions and cause the accumulation of virions in the apical region of the cells (Johnson et al., 2001). Interestingly, mutations in gE and gI yield plaques that are smaller in size as compared to WT virus in epithelial cell monolayers (Dingwell et al., 1994). Again, as mentioned above alterations in the distribution of virions within drug-treated cells can be analyzed, among others by confocal microscopy with different reagents, including viruses that encode fluorescent reporter genes fused to structural viral elements (Sugimoto et al., 2008).

## Concluding remarks

Thanks to decades of research on herpes simplex viruses, the implementation of experimental methods aimed at assessing different steps involved in the infectious cycles of these viruses, as well as the availability of different strategies to narrow down the effects of a particular drug in the replication cycle of a virus, it is now possible to use such knowledge to quickly determine at which step drugs exert their antiviral effects against HSVs. Indeed, the experimental approaches described above, which are simple and fast to carry out, should help identify potential mechanisms of action of antivirals, which in turn may contribute at identifying and designing better drugs against HSV directed to viral proteins or by the modulation of cellular targets that make cells resistant to HSV infection (Ibáñez et al., 2017; Jaishankar et al., 2018). Alternatively, carrying out further experimentation on top of the assays described above will contribute with more detail on particular viral infection steps and potentially identify new phases in the HSV replication cycle, altogether providing new opportunities for discovering and designing better drugs against these viruses. Overall, the more we know about the mechanisms of action of drugs against HSV, the closer we will be to improve therapies against these viruses that cause important burden in humans.

## Author contributions

FI, MF, MG-T, NC, LD, AR-D, and PG wrote the manuscript and designed the figures. All authors reviewed the manuscript.

### Conflict of interest statement

The authors declare that the research was conducted in the absence of any commercial or financial relationships that could be construed as a potential conflict of interest.
